# Twist-mediated Epithelial-mesenchymal Transition Promotes Breast Tumor Cell Invasion via Inhibition of Hippo Pathway

**DOI:** 10.1038/srep24606

**Published:** 2016-04-20

**Authors:** Yifan Wang, Jingyi Liu, Xuhua Ying, Pengnian Charles Lin, Binhua P. Zhou

**Affiliations:** 1 Cancer Institute of Integrative Medicine, Zhejiang Academy of Chinese Medicine, Hangzhou, Zhejiang, 310007, China; 2 The State Key Laboratory of Experimental Hematology, Institute of Hematology and Blood Diseases Hospital, Chinese Academy of Medical Sciences and Peking Union Medical College, Tianjin 300020, China; 3 Center for Cancer Research, National Cancer Institute-Frederick, Frederick, MD 21702, USA; 4 Department of Molecular and Cellular Biochemistry, and Markey Cancer Center, the University of Kentucky, College of Medicine, Lexington, KY 40506, United States

## Abstract

Twist is a key transcription factor for Epithelial-mesenchymal transition (EMT), which is a cellular de-differentiation program that promotes invasion and metastasis, confers tumor cells with cancer stem cell (CSC)-like characteristics, and increases therapeutic resistance. However, the mechanisms that facilitate the functions of Twist remain unclear. Here we report that Twist overexpression increased expression of PAR1, an upstream regulator of the Hippo pathway; PAR1 promotes invasion, migration, and CSC-like properties in breast cancer by activating the transcriptional co-activator TAZ. Our study indicates that Hippo pathway inhibition is required for the increased migratory and invasiveness ability of breast cancer cells in Twist-mediated EMT.

Breast cancer is the most common cancer in women worldwide, and approximately 90% of breast cancer deaths are the result of metastasis. Metastasis is the process by which tumor cells detach from a primary tumor and migrate to nearby blood vessels or the lymph system, and are thereby able to spread to other organs in the host^1^. During metastasis, tumor cells acquire a highly motile phenotype through a de-differentiation program known as epithelial to mesenchymal transition (EMT). EMT, a phenomenon traditionally associated with embryonic development, is now accepted as a central mechanism that induces invasion and metastasis of tumors^2^,^3^. As part of the EMT process, epithelial cells lose their apical-basal polarity and intercellular adhesive property; in proxy, the cells gain mesenchymal properties, including fibroblast-like morphology and increased motility, all of which favor invasion and dissemination. EMT also bestows tumor cells with cancer stem cell (CSC)-like characteristics, and an associated therapeutic resistance.

Breast cancer is a heterogeneous disease in terms of tumor histology, clinical presentation, and response to therapy. There are four major subtypes based on gene expression profiling: luminal A, luminal B, ErbB2, and basal like. Breast cancer undergoes EMT and show a basal-like phenotype, suggesting that EMT occurs within a specific genetic context in breast cancers^4^. A better understanding of the mechanisms that support the EMT program in breast cancer is crucial in order to develop new therapeutic strategies. A hallmark of EMT is the loss of E-cadherin expression^3^. Several transcription factors have been implicated in the transcriptional repression of E-cadherin and function as molecular switches for the EMT program^3^,^5^,^6^. Twist and Snail are two transcriptional factors that are crucial to EMT activation, and cooperate to support development of full invasive and metastatic capacity. For example, during the mesoderm formation in *Drosophila*, twist and snail function as positive and negative regulators, respectively; Twist acts as a transcriptional activator to induce mesodermal gene expression, whereas Snail serves as a transcriptional suppressor to prevent expression of genes that belong to ectoderm^7^. Similar cooperative activities suggest that Twist and Snail work synergistically to induce EMT^8^.

Protease-activated receptors (PARs) are members of a subfamily of G protein-coupled receptors that play critical roles in development, inflammation and angiogenesis, and cancer. PARs contribute to tumor cell motility and metastasis^9^. PAR1, also known as the coagulation factor II (thrombin) receptor, is a protein encoded by the *F2R* gene in humans. PAR1 is thought to be involved in the invasive and metastatic processes of several types of cancer, including breast, colon, lung, pancreas and prostate cancers^10^,^11^,^12^,^13^. Recent research shows that the PARs are upstream signals of Hippo pathway^14^. The Hippo signaling pathway, initially discovered by genetic studies in *Drosophila* as a regulator of organ size, plays a crucial role in tissue growth, and in tumorigenesis^15^. PAR1 acts through the G_12/13_ and Rho GTPase to inhibit the Hippo pathway kinases Lats1/2; this kinase activates downstream signaling of YAP/TAZ by decreasing its phosphorylation and increasing nuclear localization^14^. Therefore, Hippo inhibition and the associated YAP/TAZ activation function as a key downstream signaling branch of PAR1 activation. However, the proteases responsible for activating the pro-invasive functions of PAR1 are, to date, not identified.

In this study, we found that the expression of Twist induced EMT in mammary epithelial cells and luminal breast cancer cells, and that PAR1 and TAZ were activated in these Twist-overexpressing transfectants. Knockdown of TAZ expression significantly decreased the expression of connective tissue growth factor (CTGF) and suppressed the invasive properties mediated by Twist. Together, our results indicate that the activation of PAR1 and the inhibition of Hippo pathway are required for the Twist-induced EMT. Therefore, our study not only reveals a critical mechanism underlying metastasis but also has implications for the development of therapeutic strategies for breast cancer.

## Results

### Overexpression of Snail or Twist induces EMT

**Figure 1:**
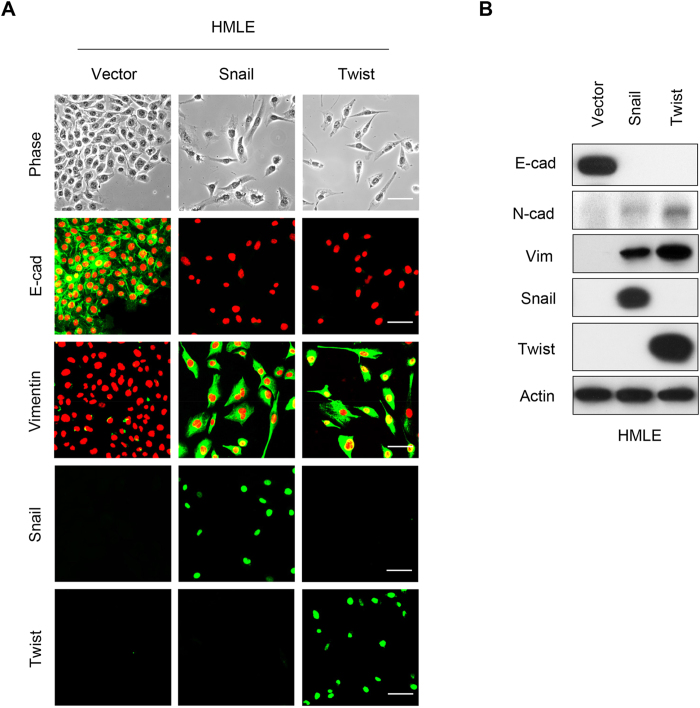
Overexpression of Snail or Twist induces EMT in HMLE cells. (**A**) Representative images show expression of E-cadherin and Vimentin in Snail- or Twist-expressing HMLE cells analyzed by immunofluorescent staining. Nuclei were visualized with DAPI staining (red). The morphologic changes associated with EMT are shown in the representative phase contrast images. Scale bars, 50 μm. (**B**) Expression of E-cadherin, N-cadherin and Vimentin in these cells was assessed by western blot analysis; actin served as a loading control.

**Figure 2:**
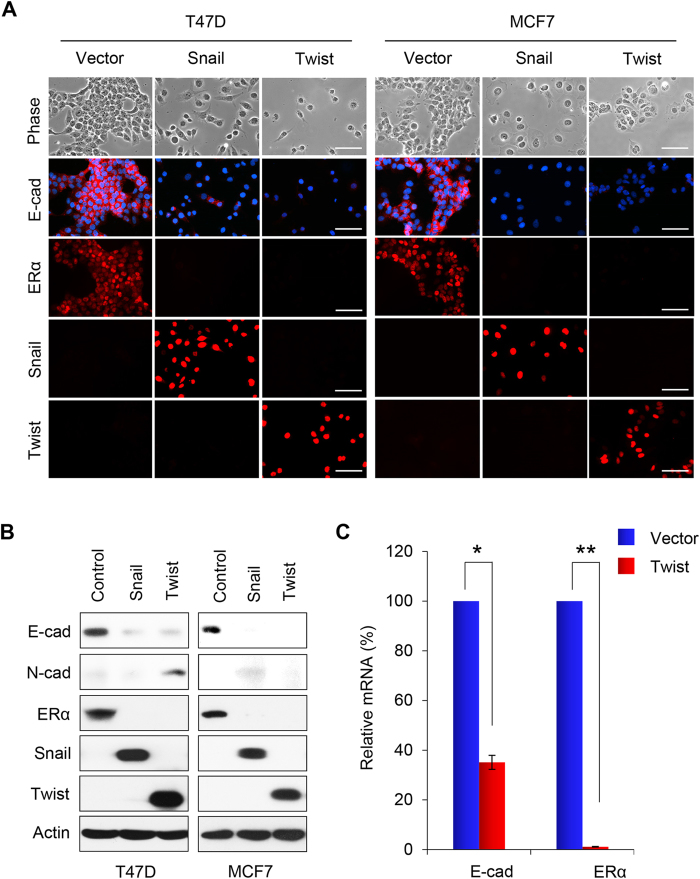
Overexpression of Snail or Twist induces EMT in T47D and MCF7 cells. (**A**) Representative images show expression of E-cadherin and ERα in Snail- or Twist-expressing T47D and MCF7 cells analyzed by immunofluorescent staining. Nuclei were visualized with DAPI staining (blue). The morphologic changes associated with EMT are shown in representative phase contrast images. Scale bars, 50 μm. (**B**) Expression of E-cadherin, N-cadherin and ERα in these cells was assessed by western blot analysis; actin served as a loading control. **(C)** Quantification of the relative mRNA levels of E-cadherin and ERα in Twist expressing T47D cells compared with vector-control cells using real-time PCR. Presented data are the mean ± SD from three separate experiments, with *and **indicate p < 0.01 in comparison with that of control.

To determine the role of Snail and Twist in EMT, we expressed Snail or Twist in immortalized human mammary epithelial cells (HMLE). Expression of Snail or Twist induced morphologic changes in HMLE cells, from a cobble-stone-like epithelial appearance to a spindle-shaped fibroblastic-like phenotype; these cells became elongated in shape and disassociated from their neighboring cells (Fig. 1A). Immunofluorescence staining showed downregulation of the epithelial marker E-cadherin, and upregulation of the mesenchymal marker Vimentin. Western blot analysis confirmed these results (Fig. 1B). We also expressed Snail or Twist in two luminal breast cancer cell lines, T47D and MCF7, that contain little endogenous Snail and Twist. Expression of Snail or Twist induced EMT in these cells, and converted the morphology of luminal cells to a basal-like phenotype (Fig. 2A). In addition, we found downregulation of the luminal epithelial markers E-cadherin and ERα, and the upregulation of the mesenchymal marker N-cadherin by immunofluorescence and western blot analysis (Fig. 2A,B). Long term (over 10 days) expression of Snail in T47D and MCF7 cells led to apoptosis in both cell lines (Table 1), and expression of Twist in MCF7 cells also led to apoptosis in this cell line. Interestingly, overexpression of Twist in T47D cells did not result in apoptosis, but led to the formation of a stable cell line with morphologic changes typical of EMT (Table 1). The mRNA levels of E-cadherin and ERα were dramatically decreased in this transformed cell line (Fig. 2C).

### Twist expands the stem cell population

**Figure 3:**
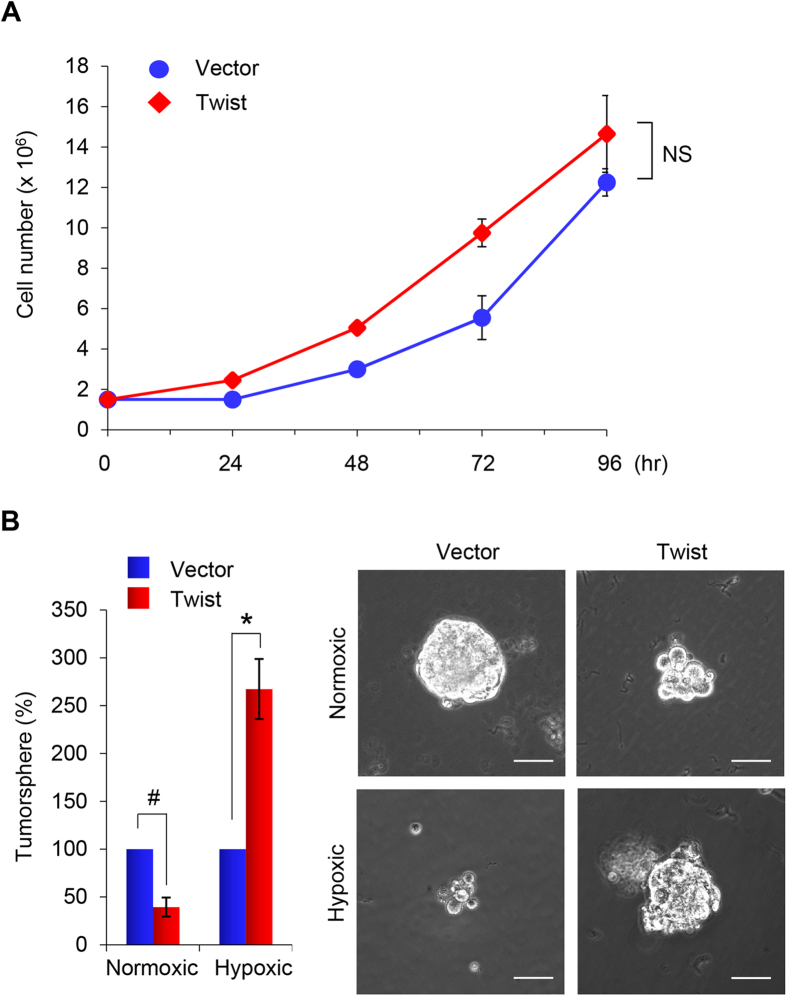
Overexpression of Twist induces CSC-like properties in T47D cells. (**A**) Graphic representation of cell growth rates by T47D cells stably expressing Twist or control vector. Cell counts were obtained daily over a 4 day period. Presented data are the mean ± SD from two independent experiments with triplicate samples. NS stands for statistically non-significant. (**B**) Tumorsphere formation was assessed in T47D cells overexpressing Twist under normoxic or hypoxic conditions. Representative images of tumorspheres are shown in the right panel. Scale bars, 100 μm. Left panel, are graphic representations of tumorsphere number. Presented data are the percentage of control vector values, with mean ± SD of three separate experiments performed in duplicate. ^#^p <  0.05 and *p <  0.01 when vector control cells compared with their Twist-expressing clones, respectively.

To investigate whether Twist-expression affects proliferation of breast cancer cells, we measured cell growth of T47D-Twist cells by cell counting. T47D-Twist cells did not demonstrate a significant growth difference compared with the vector control cells over the 96-hour interval examined (Fig. 3A). We also examined tumorsphere formation of these cells, which is based on the unique property of stem/progenitor cells to survive and grow in serum-free suspension. Although both T47D-vector and T47D-Twist cell types did form tumorspheres, the size and density of tumorspheres formed by T47D-Twist cells were lightly smaller than those formed by vector control cells under normoxic conditions. Under hypoxic conditions, tumorspheres were sparse in vector control cells. Surprisingly, the size and density of tumorsphere formed by T47D-Twist cells were much bigger than that of control cells (Fig. 3B). These results suggest that expression of Twist promotes induction of CSC-like properties and their growth in T47D cells under hypoxic conditions.

### Twist promotes cell migration and invasion

**Figure 4:**
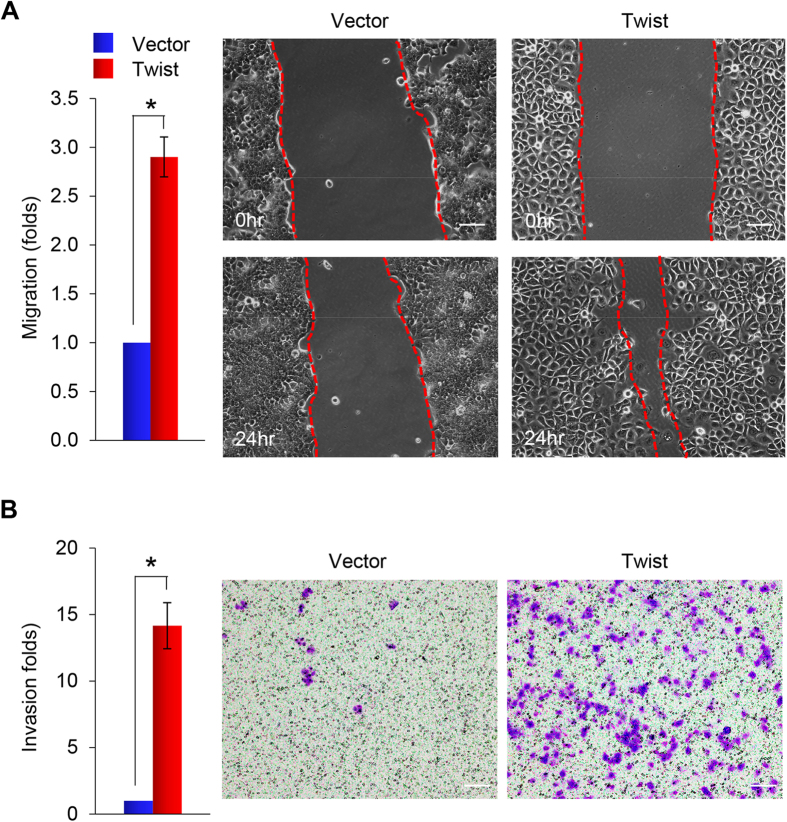
Overexpression of Twist enhances cell migration and invasion of T47D cells. (**A**) Graphic representation of the migratory capability of stably transfected T47D cells expressing either Twist or control vector assessed using a wound healing assay. A scratch (“wound”) was inflicted to a cell layer produced 48 hours post-plating, and culture continued for an additional 24 hrs. Wound closures were photographed at 0 and 24 hr. Presented data are the mean ± SD from three independent experiments, with *indicates p < 0.01 when comparing with control values. A representative experiment is shown in the right panel. Scale bars, 50 μm. (**B**) Graphic representation of the invasiveness of T47D cells stably expressing Twist or control vector using a modified Boyden Chamber invasion assay as described in the Materials and Methods. Presented data are the mean ± SD from three separate experiments, with *indicates p < 0.01 when comparing with control values. A representative experiment is shown in the right panel. Scale bars, 100 μm.

To investigate the migratory and invasive capabilities mediated by Twist, we performed an *in vitro* wound healing assay, which is commonly used to assess the effects of exogenous gene expression on the migration of individual cells. Closure of the scratch wound required significantly less time in T47D-Twist cells than in vector control cells (Fig. 4A). Statistical analysis indicated that migration activity of T47D-Twist cells was about 3-fold higher than that of vector control cells (Fig. 4A). We also used Matrigel-coated Boyden chambers to assess cell invasiveness; the invasion capacity of T47D-Twist cells increased 14-fold compared with that of vector control cells (Fig. 4B).

### Twist induces PAR1 activation and Hippo inhibition

**Figure 5:**
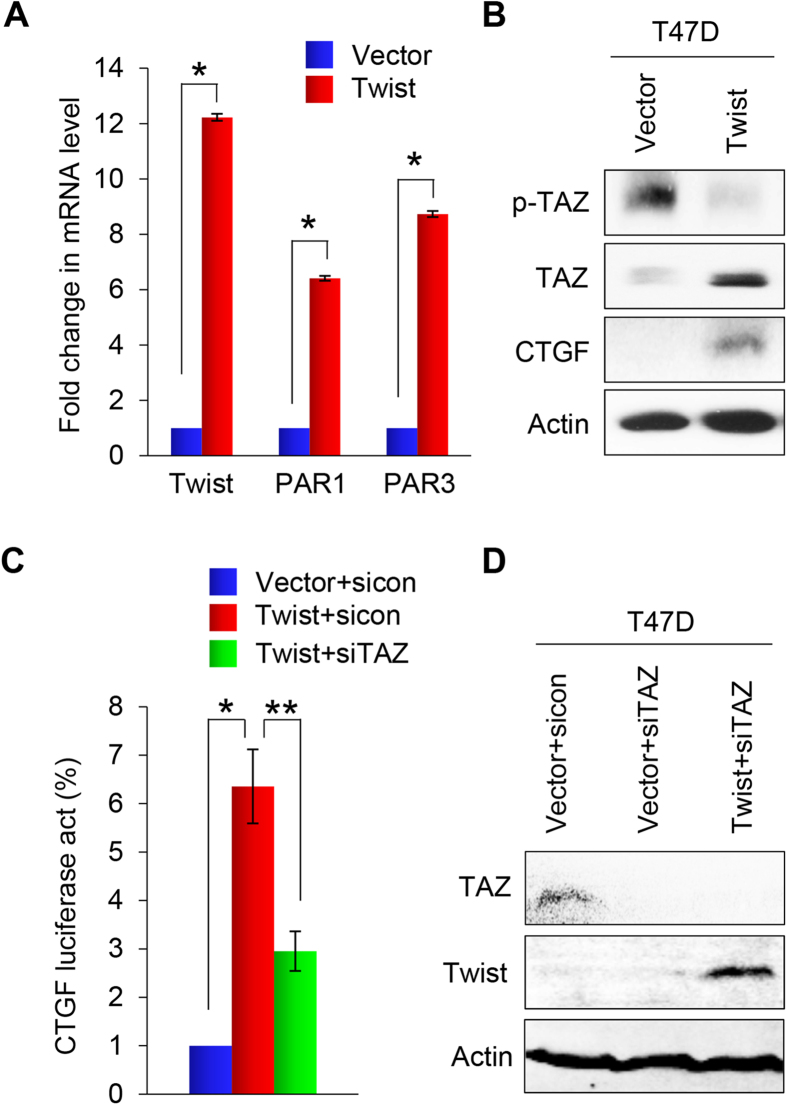
Overexpression of Twist induces the activation of PAR1 signaling. (**A**) Graphic representation of the fold change in mRNA levels of Twist, F2R and F2RL2 in Twist-expressing T47D cells compared with control vector cells by real-time PCR. Presented data are the mean ± SD of three separate experiments, with *indicates p < 0.01 when comparing with control values. (**B**) Western blot analysis for p-TAZ, TAZ and CTGF expression in EMT-induced Twist-expressing T47D cells or T47D cells expressing control vector. Actin served as a loading control. (**C**) Effect of TAZ siRNA or NTC siRNA on CTGF promoter luciferase activity in Twist-overexpressing T47D cells and T47D cells expressing control vector. Assessments were made after 48 hours in culture. Presented data are mean ± SD of normalized luciferase activities determined from three separate experiments. *indicates p < 0.01 when control siRNA expressed in Twist-T47D cells compared with in vector control cells; and **indicates p < 0.01 when compared expression of TAZ siRNA and control siRNA in Twist-T47D cells. (**D**) Effect of TAZ siRNA on TAZ and Twist expression in EMT-induced Twist-expressing T47D cells and in T47D cells expressing control vector by western blot analysis. Actin served as a loading control.

To understand the molecular mechanisms associated with Twist-induced EMT, we performed cDNA microarray analysis of HMLE and T47D cells that had undergone Twist-mediated EMT (Figs 1A and 2A). The mRNA of two PARs family genes, PAR1 (*F2R*) and PAR3 (*F2RL2*), were significantly elevated in both cell lines. These results were confirmed by qRT-PCR (Fig. 5A). Recently, these PAR proteins were identified as upstream regulators of the Hippo pathway, and play a crucial role in breast cancer invasion and metastasis. These data suggest that Twist regulates the Hippo pathway by upregulating PAR expression.

Next, we investigated whether Twist expression suppresses the Hippo pathway by examing the expression of TAZ in T47D-Twist cells and the corresponding control vector cells. Since PAR1 inhibits the Lats1/2 kinases, we would expect that a Twist-mediated increase in PAR1 expression would decrease TAZ phosphorylation and increase TAZ activity. We found that TAZ was activated in T47D-Twist cells, and that the level of phosphorylated TAZ was significantly reduced (Fig. 5B). Consistent with this, the expression level of CTGF, a downstream transcriptional target of TAZ, was increased (Fig. 5B). These results support the idea that Twist suppresses the Hippo pathway by upregulating the PAR1 signaling.

**Figure 6:**
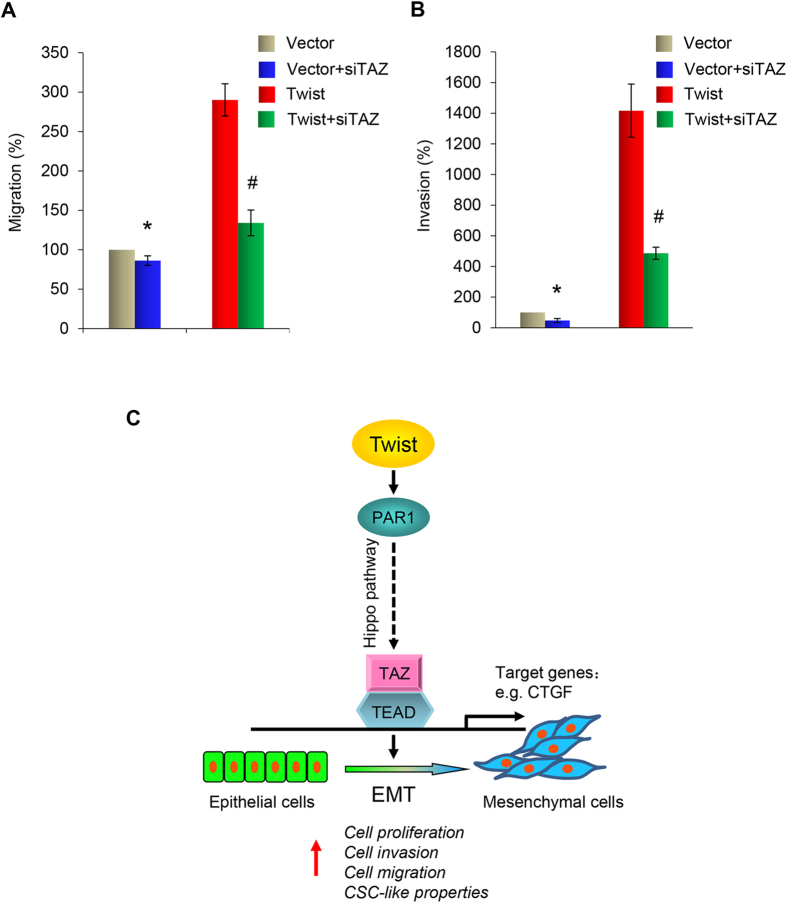
Knockdown of TAZ suppresses Twist-induced cell migration and invasion of T47D cells. (**A**) Effect of TAZ siRNA on cell migrating activity in Twist-overexpressing T47D cells and T47D cells expressing control vector using a wound healing assay. A scratch (“wound”) was inflicted to a cell layer produced 48 hours post-plating, and culture continued for an additional 24 hrs. Wound closures were photographed at 0 and 24 hr. Presented data are the mean ± SD from three independent experiments, with *and ^#^indicating significant difference of p < 0.05 from control values. **(B)** Effect of TAZ siRNA on cell invasiveness in Twist-overexpressing T47D cells and T47D cells expressing control vector using a modified Boyden Chamber invasion assay as described in the Materials and Methods. Presented data are a graphic representation of the mean ± SD of percentage of invasive cells obtained from three separate experiments, with *and ^#^indicating significant difference of p < 0.05 from control values. **(C)** Expression of Twist results in increased expression of PAR1, which promotes invasion, migration, and induces CSC-like properties in breast cancer cells by upregulating the expression of TAZ.

If TAZ activation is crucial for the Twist-mediated EMT, depletion of TAZ should reverse the changes induced by Twist. To test this possibility, we employed a luciferase reporter assay to determine if TAZ is required for the Twist-mediated activation of CTGF promoter. We found that expression of Twist increased the promoter activity of CTGF, however, this effect was blocked by knocking down the expression of TAZ (Fig. 5C). Taken together, these results indicate that Twist expression activates TAZ, which leads to an increase in CTGF promoter transactivation as measured by luciferase activity. To further confirm the effect of TAZ, we knocked down the expression of TAZ by siTAZ in T47D-Twist cells and control vector cells (Fig. 5D), and performed wound healing and invasion analyses. We found that the migration induced by Twist in T47D cells was reduced from 3-fold to 1.3-fold (Fig. 6A), and that the invasion capability was reduced from 14-fold to 5-fold (Fig. 6B); these data indicate that Hippo pathway inhibition is required for the increased migratory and invasiveness in Twist-mediated EMT.

## Discussion

Accumulating evidence indicates that EMT-associated transcription factors endow cells with malignant traits, such as invasion, migration and therapeutic resistance. In this study, we showed that the exogenous expression of Twist induced EMT not only in normal mammary epithelial cells HMLE, but also in the luminal breast cancer cells T47D. We established a T47D cell line stably expressing Twist, and this cell line displayed a mesenchymal cell phenotype.

Our study demonstrates that Twist-induced EMT in T47D cells is accompanied by functional effects, including increased cell invasion, migration and CSC-like properties under hypoxic conditions. We also established that Twist activates PAR1 and PAR3 gene expressions. The PARs are a unique class of G-protein-coupled receptors that act as high-gain sensors of extracellular protease gradients, allowing cells to react to a proteolytic microenvironment^10^. PARs have been implicated in tumor progression. PAR1 is not expressed in normal breast epithelia but is upregulated in invasive breast carcinomas^16^. The invasive MDA-MB-231 breast cancer cell line express high levels of PAR1^17^. In our study, activation of PAR1 in noninvasive T47D cells accompanied the acquisition of a mesenchymal phenotype and suppression of the Hippo pathway (Fig. 6C).

Major components of the Hippo pathway, including the MST1/2 and Lats1/2 kinases, are highly conserved and play an important role in control of mammalian organ size, cell proliferation, apoptosis, and stem cell differentiation^18^. PARs regulate the Hippo pathway. Activation of PAR1 and other G protein coupled receptors that link to G_12/13_, trigger the activation of Rho GTPase, which works through the actin cytoskeleton to inhibit the Lats1/2 kinase and subsequently activates YAP/TAZ by decreasing its phosphorylation and increasing nuclear localization^14^. PAR3 was speculated to modulate PAR1 signaling by receptor dimerization. PAR3 had been reported functions as an important allosteric modulator of PAR1 signaling. PAR1 heterodimerization with PAR3 alters the PAR1/Gα_13_ binding conformation, enhancing Gα_13_ signaling^19^. TAZ, a transducer of Hippo pathway, was reported to confer CSC-related traits on breast cancer cells, and was required to sustain self-renewal and tumor-initiation capacities in breast cancer stem cells^20^. We hypothesize that PAR1 overexpression induced by Twist could contribute to breast cancer cell progression by inhibiting Hippo pathway. In our study, we found that the expression level of TAZ was upregulated by Twist expression, while the phosphorylation level of TAZ was downregulated. Dephosphorylated TAZ functions as transcription coactivators for the TEAD family of transcription factors to induce gene expression, thereby promoting cell growth, proliferation, and survival^21^,^22^,^23^. We found that the expression level of CTGF, a known TAZ target gene^15^,^24^, was increased in Twist-expressing T47D cells. The luciferase activity of the CTGF promoter was also enhanced. To further address the role of TAZ in the Twist regulated Hippo pathway, we knocked down the expression of TAZ, and found that clear reversion of luciferase activity was observed by TAZ depletion in Twist-expressing T47D cells. Moreover, TAZ ablation suppressed migration and invasion capability as determined by the wound healing and invasion assays. These results imply that Twist activates TAZ in the Hippo pathway and that TAZ depletion inhibits Twist-induced cell migration and invasion.

In summary, we delineated the cross-talk between Twist-mediated EMT and the Hippo pathway in metastatic breast cancer. We showed that PAR1 was activated by Twist. The induction of PAR1 expression was critical for the Twist-mediated promotion of EMT and cell invasion and migration. Activated PAR1 signaling induced the expression of TAZ by suppressing Hippo pathway, and bestowed breast cancer cells with stem cell properties (Fig. 6C). Together, our experiments revealed a role for PAR1 in promoting EMT and invasive potential of breast cancer cells, suggesting that PAR1 might be a potential therapeutic target for EMT and metastasis of breast cancer.

## Methods

### Plasmids, siRNA, and Antibodies

Smart pool siRNA against TAZ was obtained from Dharmacon (Chicago, IL). Human Twist and Snail were amplified from a HeLa cDNA library and subcloned into pLenti6.3/V5. Antibodies for Vimentin and ERα were from Neomarkers (Fremont, CA). Antibodies for E-cadherin and TAZ were from BD Transduction Laboratories (San Jose, CA). Antibodies for Twist, YAP, p-YAP and CTGF were purchased from Santa Cruz Biotechnology (Santa Cruz, CA). Snail and N-cadherin antibodies was purchased from Cell Signaling Technology (Danvers, MA) and Upstate (Charlottesville, VA), respectively.

### Cell Culture

The MCF7 breast cancer cell line was grown in Dulbecco’s modified Eagle’s medium (DMEM)/F12 supplemented with 10% fetal bovine serum (FBS). T47D cells were grown in RPMI1640 plus 10% FBS. HMLE was grown in DMEM/F-12 medium plus 10 ng/mL EGF, 10 μg/mL insulin and 0.5 μg/mL hydrocortisone. For establishing stable clones, transfected breast cell lines were selected with puromycin (1 μg/mL) for 4 wks.

### Immunostaining and Immunoblotting

Experiments were performed as described previously^25^,^26^. For immunofluorescent staining, cells grown on chamber slides, were fixed with 4% paraformaldehyde, and incubated overnight with primary antibodies. Secondary antibodies were Alexa Fluor 488 goat anti-mouse IgG (H + L), Alexa Fluor 568 goat anti-mouse IgG (H + L), or Alexa Fluor 568 goat anti-rat IgG (H + L) (Molecular Probe, Carlsbad, CA).

### Quantitative Real-Time PCR

Total RNA was prepared using the RNeasy Mini kit (Qiagen) according to the manufacturer’s instructions. Specific quantitative real-time PCR experiments were performed using SYBR Green Power Master Mix following manufacturer’s protocol (Applied Biosystems).

### Luciferase Reporter Assay

Luciferase reporter assays were performed as described previously^25^,^27^. Cells grown to 50% confluence in six-well plates were co-transfected with reporter gene constructs using Fugene 6 (Roche, Indianapolis, IN). To normalize transfection efficiency, cells were also co-transfected with pTK-RL (Renilla luciferase). Cell extracts, prepared 48 hrs after transfection, were assessed for luciferase activity using the Dual-Luciferase Reporter Assay System (Promega, Madison, WI). All experiments were performed three times in triplicate.

### Invasion Assay

Invasion assays were performed in Boyden chambers coated with Matrigel as instructed by the manufacturer (BD Biosciences, San Jose, CA). Cancer cells were seeded on top of the Matrigel in the upper chamber, and the bottom chamber was filled with culture medium containing EGF (10 ng/mL) as the chemoattractant. The invasive cancer cells, on the underside of the Boyden chamber membrane, were fixed with paraformaldehyde, stained with crystal violet and counted. All experiments were performed in triplicate.

### Tumorsphere Assay

Tumorsphere assays were performed following the protocol previously described^28^,^29^. Briefly, cells were seeded in single-cell suspension in triplicate into ultra-low attachment 6-well plates (Corning) in DMEM/F12 medium supplemented with 20 ng/mL EGF, 5 mg/mL insulin, 0.5 mg/mL hydrocortisone and 2% B27. After 1 to 2 wks incubation, the presence of spheres (3D multicellular structures greater than 40 μm in diameter) was assessed by inverted microscopy. Ten random fields for each cell lines were visualized; the number and size of spheres in the 10 fields were calculated as a percentage over that of parent cells.

### Statistical Analysis

Experiments were repeated at least twice. Data are presented as mean ± SD. A Student’s t-test (two tailed) was used to compare two groups. p < 0.05 was considered statistically significant.

## Additional Information

**How to cite this article**: Wang, Y. *et al.* Twist-mediated Epithelial-mesenchymal Transition Promotes Breast Tumor Cell Invasion via Inhibition of Hippo Pathway. *Sci. Rep.* **6**, 24606; doi: 10.1038/srep24606 (2016).

## Figures and Tables

**Table 1:** EMT, apoptosis and survival status in T47D and MCF7 cells with Snail/Twist overexpression.

Cell lines		EMT	Apoptosis	Survival
T47D	control	No	No	Yes
	Snail	Yes	Yes	No
	Twist	Yes	No	Yes
MCF7	control	No	No	Yes
	Snail	Yes	Yes	No
	Twist	No	Yes	No

## References

[b1] Pantel K. & Brakenhoff R. H. Dissecting the metastatic cascade. Nat Rev Cancer. 4, 448–456 (2004). 15170447 10.1038/nrc1370

[b2] Polyak K. & Weinberg R. A. Transitions between epithelial and mesenchymal states: acquisition of malignant and stem cell traits. Nat Rev Cancer. 9, 265–273 (2009). 19262571 10.1038/nrc2620

[b3] Thiery J. P., Acloque H., Huang R. Y. & Nieto M. A. Epithelial-mesenchymal transitions in development and disease. Cell. 139, 871–890 (2009). 19945376 10.1016/j.cell.2009.11.007

[b4] Sarrio D. *et al.* Epithelial-mesenchymal transition in breast cancer relates to the basal-like phenotype. Cancer Res. 68, 989–997 (2008). 18281472 10.1158/0008-5472.CAN-07-2017

[b5] Kalluri R. & Weinberg R. A. The basics of epithelial-mesenchymal transition. J Clin Invest. 119, 1420–1428 (2009). 19487818 10.1172/JCI39104 PMC2689101

[b6] Yang J. *et al.* Twist, a master regulator of morphogenesis, plays an essential role in tumor metastasis. Cell. 117, 927–939 (2004). 15210113 10.1016/j.cell.2004.06.006

[b7] Leptin M. twist and snail as positive and negative regulators during Drosophila mesoderm development. Genes Dev. 5, 1568–1576 (1991). 1884999 10.1101/gad.5.9.1568

[b8] Zeitlinger J. *et al.* Whole-genome ChIP-chip analysis of Dorsal, Twist, and Snail suggests integration of diverse patterning processes in the Drosophila embryo. Genes Dev. 21, 385–390 (2007). 17322397 10.1101/gad.1509607 PMC1804326

[b9] Shi X., Gangadharan B., Brass L. F., Ruf W. & Mueller B. M. Protease-activated receptors (PAR1 and PAR2) contribute to tumor cell motility and metastasis. Mol Cancer Res. 2, 395–402 (2004). 15280447

[b10] Boire A. *et al.* PAR1 is a matrix metalloprotease-1 receptor that promotes invasion and tumorigenesis of breast cancer cells. Cell. 120, 303–313 (2005). 15707890 10.1016/j.cell.2004.12.018

[b11] Nierodzik M. L., Kajumo F. & Karpatkin S. Effect of thrombin treatment of tumor cells on adhesion of tumor cells to platelets *in vitro* and tumor metastasis *in vivo*. Cancer Res. 52, 3267–3272 (1992). 1596884

[b12] Heider I. *et al.* PAR1-type thrombin receptor stimulates migration and matrix adhesion of human colon carcinoma cells by a PKCepsilon-dependent mechanism. Oncol Res. 14, 475–482 (2004). 15559761 10.3727/0965040042380496

[b13] Even-Ram S. *et al.* Thrombin receptor overexpression in malignant and physiological invasion processes. Nat Med. 4, 909–914 (1998). 9701242 10.1038/nm0898-909

[b14] Mo J. S., Yu F. X., Gong R., Brown J. H. & Guan K. L. Regulation of the Hippo-YAP pathway by protease-activated receptors (PARs). Genes Dev. 26, 2138–2143 (2012). 22972936 10.1101/gad.197582.112 PMC3465735

[b15] Pan D. The hippo signaling pathway in development and cancer. Dev Cell. 19, 491–505 (2010). 20951342 10.1016/j.devcel.2010.09.011 PMC3124840

[b16] Yang E. *et al.* Blockade of PAR1 signaling with cell-penetrating pepducins inhibits Akt survival pathways in breast cancer cells and suppresses tumor survival and metastasis. Cancer Res. 69, 6223–6231 (2009). 19622769 10.1158/0008-5472.CAN-09-0187 PMC2733168

[b17] Kamath L., Meydani A., Foss F. & Kuliopulos A. Signaling from protease-activated receptor-1 inhibits migration and invasion of breast cancer cells. Cancer Res. 61, 5933–5940 (2001). 11479236

[b18] Zhao B., Li L., Lei Q. & Guan K. L. The Hippo-YAP pathway in organ size control and tumorigenesis: an updated version. Genes Dev. 24, 862–874 (2010). 20439427 10.1101/gad.1909210 PMC2861185

[b19] McLaughlin J. N., Patterson M. M. & Malik A. B. Protease-activated receptor-3 (PAR3) regulates PAR1 signaling by receptor dimerization. Proc Natl Acad Sci USA 104, 5662–5667 (2007). 17376866 10.1073/pnas.0700763104 PMC1838494

[b20] Cordenonsi M. *et al.* The Hippo transducer TAZ confers cancer stem cell-related traits on breast cancer cells. Cell. 147, 759–772 (2011). 22078877 10.1016/j.cell.2011.09.048

[b21] Lei Q. Y. *et al.* TAZ promotes cell proliferation and epithelial-mesenchymal transition and is inhibited by the hippo pathway. Mol Cell Biol. 28, 2426–2436 (2008). 18227151 10.1128/MCB.01874-07 PMC2268418

[b22] Zhao B. *et al.* TEAD mediates YAP-dependent gene induction and growth control. Genes Dev. 22, 1962–1971 (2008). 18579750 10.1101/gad.1664408 PMC2492741

[b23] Zhao B., Li L., Tumaneng K., Wang C. Y. & Guan K. L. A coordinated phosphorylation by Lats and CK1 regulates YAP stability through SCF(beta-TRCP). Genes Dev. 24, 72–85 (2010). 20048001 10.1101/gad.1843810 PMC2802193

[b24] Lai D., Ho K. C., Hao Y. & Yang X. Taxol resistance in breast cancer cells is mediated by the hippo pathway component TAZ and its downstream transcriptional targets Cyr61 and CTGF. Cancer Res. 71, 2728–2738 (2011). 21349946 10.1158/0008-5472.CAN-10-2711

[b25] Wu Y. *et al.* Stabilization of snail by NF-kappaB is required for inflammation-induced cell migration and invasion. Cancer Cell. 15, 416–428 (2009). 19411070 10.1016/j.ccr.2009.03.016 PMC2881229

[b26] Zhou B. P. *et al.* Dual regulation of Snail by GSK-3beta-mediated phosphorylation in control of epithelial-mesenchymal transition. Nat Cell Biol. 6, 931–940 (2004). 15448698 10.1038/ncb1173

[b27] Dong C. *et al.* G9a interacts with Snail and is critical for Snail-mediated E-cadherin repression in human breast cancer. J Clin Invest. 122, 1469–1486 (2012). 22406531 10.1172/JCI57349 PMC3314447

[b28] Grimshaw M. J. *et al.* Mammosphere culture of metastatic breast cancer cells enriches for tumorigenic breast cancer cells. Breast Cancer Res. 10, R52 (2008). 18541018 10.1186/bcr2106 PMC2481500

[b29] Smart C. E. *et al.* *In vitro* analysis of breast cancer cell line tumourspheres and primary human breast epithelia mammospheres demonstrates inter- and intrasphere heterogeneity. PLoS One. 8, e64388 (2013). 23750209 10.1371/journal.pone.0064388 PMC3672101

